# Estrogen-regulated miRNA-27b is altered by bisphenol A in human endometrial stromal cells

**DOI:** 10.1530/REP-18-0041

**Published:** 2018-09-28

**Authors:** Beverly G Reed, Samir N Babayev, Lucy X Chen, Bruce R Carr, R Ann Word, Patricia T Jimenez

**Affiliations:** Division of Reproductive Endocrinology and Infertility Department of Obstetrics and Gynecology, Green Center for Reproductive Biological Sciences, University of Texas Southwestern Medical Center, Dallas, Texas, USA

## Abstract

MicroRNAs (miRs) are small molecules important for regulation of transcription and translation. The objective was to identify hormonally regulated miRs in human endometrial stromal cells and to determine the impact of the endocrine disruptor, bisphenol A (BPA), on those miRs. miR microarray analysis and multiple confirmatory cell preparations treated with 17β-estradiol (E2) and BPA altered miR-27b, let-7c, let-7e and miR-181b. Further, decidualization downregulated miR-27b. *VEGFB* and *VEGFC* were validated as targets of miR-27b. Identification of miR-27b target genes suggests that BPA and E2 downregulate miR-27b thereby leading to upregulation of genes important for vascularization and angiogenesis of the endometrium during the menstrual cycle and decidualization.

## Introduction

Endometrial development is the result of a carefully coordinated set of events largely controlled by steroid hormones. 17β-estradiol (E2) mediates growth and vascularization of the endometrium. After ovulation, progesterone (P4) induces endometrial differentiation and receptivity. If implantation does not occur, progesterone levels fall resulting in endometrial shedding and menstruation. Implantation is a complex, precisely timed event, and it is an important limiting step in reproduction for many women. Implantation failures may be related to immunologic factors, endocrine or hormonal disruptions, lack of endometrial receptivity, anatomic defects (leiomyomas, intrauterine adhesions) or embryo factors (Bechi *et al.* 2010, Makker & Goel 2013, Qiong *et al.* 2017, Zhang *et al.* 2017). However, there are many cases in which a cause for implantation failure cannot be found. Whereas assisted reproductive technologies (ARTs) may overcome some cases of unexplained infertility, many women continue to have implantation failures despite transfer of good-quality euploid embryos in multiple sequential cycles (recurrent implantation failure) emphasizing that a critical aspect of achieving pregnancy in both natural and ART cycles is successful implantation. The mechanisms that regulate endometrial development and embryo implantation, and thus, implantation failure, are not clearly defined. It is hypothesized that steroid hormone-induced differentiation of endometrial cells may be impaired in not only implantation failure, but also in a number of gynecologic disorders including endometriosis, adenomyosis, abnormal uterine bleeding and endometrial adenocarcinoma.

As a hormone-responsive tissue, the endometrium is susceptible to endocrine-disrupting chemicals (EDCs). EDCs are exogenous chemicals that interfere with hormone action (Diamanti-Kandarakis *et al.* 2009). Common EDCs include bisphenol A (BPA), diethylstilbestrol (DES), dichlorodiphenyltrichloroethane (DDT) and polychlorinated biphenyl (PCB). Recently, there has been increased interest in the impact of EDCs on many aspects of human development. Exposure to EDCs may affect reproduction and increase the risk of precocious puberty and infertility (Diamanti-Kandarakis *et al.* 2009). A better understanding of how EDCs may lead to pathologic conditions is needed.

BPA, a chemical widely used in products such as plastics, thermal paper and dental sealants, has weak estrogenic activity (Krishnan *et al.* 1993, Gore 2007). Most people in industrial countries are exposed to low levels of BPA through oral ingestion or transdermal absorption (Calafat *et al.* 2008, Liao *et al.* 2012, Hormann *et al.* 2014). Animal studies have shown that low levels of BPA cause changes in behavior, brain development, the prostate gland, the mammary gland and the age at which the females attain maturity (Berger *et al.* 2007, Itoh *et al.* 2012*a*, Machtinger & Orvieto 2014). Human data are limited to epidemiologic studies. Nonetheless, BPA exposure has been linked to an increased risk for many diseases including diabetes and cardiovascular disease (Rancière *et al.* 2015, Provvisiero *et al.* 2016). It may also play an important role in tissue remodeling (Dairkee *et al.* 2013, Hwang *et al.* 2013, Dong *et al.* 2014, Chen *et al.* 2015, Nakano *et al.* 2016). In regard to fertility, BPA may affect reproduction in multiple ways including uterine/endometrial factors, ovarian factors, semen parameters and implantation (Sugiura-Ogasawara *et al.* 2005, Meeker *et al.* 2010, Peretz *et al.* 2014, Barbonetti *et al.* 2016, Forte *et al.* 2016, Ganesan & Keating 2016, Ziv-Gal & Flaws 2016, Mansur *et al.* 2017). Souter *et al.* observed an inverse relationship between urinary BPA concentration and the ovarian antral follicle count (a commonly used marker for ovarian reserve; Souter *et al.* 2013). In addition, women with the highest quartiles of urinary BPA concentrations had increased odds of implantation failure during *in vitro* fertilization treatment (IVF) (Ehrlich *et al.* 2012). Others have sought to understand the effect of BPA on endometrial stromal cells (Aghajanova & Giudice 2011, Forte *et al.* 2016). Because of its weak estrogenic effects, BPA may increase endometrial stromal cell proliferation. However, BPA resulted in unchanged or decreased proliferation of endometrial stromal cells (Aghajanova & Giudice 2011, Forte *et al.* 2016). Importantly, BPA exposure also may affect stromal cell differentiation and decidualization (Forte *et al.* 2016). Because endometrial stromal cell proliferation, differentiation and decidualization are critical for implantation, BPA exposure may negatively impact fertility.

MicroRNAs (miRs) are small, non-coding RNAs that bind to and functionally silence or degrade target mRNAs. There is a large and growing body of evidence demonstrating that miRs are involved in both the normal physiologic state and in pathologic gynecologic diseases such as endometriosis, leiomyomata and endometrial cancer (Kuokkanen *et al.* 2010, Snowdon *et al.* 2011, Rekker *et al.* 2013, Marsh *et al.* 2016, Nothnick *et al.* 2016, Wilczynski *et al.* 2016, Chen *et al.* 2017*a*, Logan *et al.* 2017). Therefore, our objectives were to identify hormonally regulated miRs in endometrial stromal cells and to investigate the effects of BPA on selected miRs.

## Materials and methods

### Primary human endometrial stromal cell preparation

Studies with human tissue samples were approved by University of Texas Southwestern Medical Center/Parkland Medical Health System IRB and informed consent was obtained from each woman prior to surgery. Endometrium was scraped from the uterus of reproductive aged females (18–45 years) undergoing hysterectomy for benign conditions. Patients who used exogenous hormone within the 30 days prior to surgery were excluded. Histologically confirmed proliferative phase endometrium was used for these studies. Samples were excluded if they contained any endometrial pathology such as endometrial polyps or endometrial hyperplasia.

Tissue digestion was performed for 60 min at 37°C with a solution of HBBS, collagenase type I (1 mg/mL, Sigma) and DNase I (0.1 mg/mL, Sigma). The cells were passed through a 70 μm filter to separate the epithelial glandular cells from the stromal cells. The epithelial glandular cells were discarded. The human endometrial stromal cells (HESCs) were placed in growth media (phenol-free DMEM:F12, Hepes 15 mM, 10% fetal bovine serum, 1% antibiotic/antimycotic) and cultured until 70–90% confluent. Cells were not used beyond the third passage.

### microRNA microarray analysis

HESCs were serum-starved for 24 h and then treated with vehicle (V), 3 nM E2, 100 nM P4 or a combination of 3 nM E2+ 100 nM P4 for 16 h. E2 and P4 concentrations were chosen because (i) prior studies show endometrial stromal cells are responsive at these concentrations (Casey *et al.* 1991, Itoh *et al.* 2012*b*) and (ii) these concentrations are comparable to physiologic levels (Lenton *et al.* 1982, Kettel *et al.* 1991). miRNA microarray was performed by LC Sciences with two micrograms of total RNA for each sample and run in triplicate as previously described (Renthal *et al.* 2010, Jimenez *et al.* 2016). In brief, RNA was fractionated; small RNAs were extended with poly(A) polymerase and labeled. Paraflo microfluidic chip with 2565 mature human miRNAs was used for hybridization (miRBase Sequence Database, version 6). Multiple redundant regions were included in which each region consisted of miRNA probes that detect miRNA transcripts listed in Sanger miRBase Release 21 (http://www.mirbase.org/). Multiple control probes were included on each chip. Signal intensities increased from 1 to 65,535. During data processing, signal values that deviated >50% of average values of repeating spots were discarded. Signals with *P* values <0.01 compared with background were considered detectable. After subtraction of background, data were log_2_ transformed. Datasets are available in the Gene Expression Omnibus (GEO) and Supplementary Tables 1, 2 and 3 (see section on supplementary data given at the end of this article). Biological processes enriched in estrogen-treated cells were determined using gene ontology annotation clustering using DAVID Bioinformatics Resources with *P* values <0.02 as the cut-off. The miRs that were highly expressed and found to be statistically significantly different from the vehicle or other treatment groups with a *P* value of <0.05 were chosen for further analysis.

### Hormone studies

HESCs were plated in growth media until 80% confluency. The cells were serum starved for 24 h prior to treatment with vehicle, 3 nM E2 or BPA at various concentrations (30 nM, 300 nM or 3000 nM). Cells were harvested at 4 h, 8 h, 16 h or 24 h for RNA analysis.

### Quantitative reverse transcription PCR of miRs

Total RNA was reverse transcribed using the TaqMan MicroRNA Reverse Transcription Kit (Applied Biosystems) or the TaqMan Advanced miRNA cDNA Synthesis Kit (Applied Biosystems). qPCR was performed with miR-specific TaqMan MicroRNA Assays or Taqman Advanced miRNA Assays and TaqMan Gene Expression Master Mix. The RT-qPCR was run on a 7500 Real-Time PCR System (Applied Biosystems, Foster City, CA) and normalized to U6 snRNA, miR-26a, miR-221 and/or miR-191 with the δ-δ cycle threshold method.

### RT-qPCR of mRNA expression

Reverse transcription was performed using iScript Reverse Transcription Supermix for RT-qPCR (Bio-Rad Laboratories). Primers were used with SYBR Green Master Mix to determine gene expression on a 7500 Real-Time PCR System (Applied Biosystems). Relative mRNA expression was calculated by the δ-δ cycle threshold method.

### Transfection of HESCs

HESCs were cultured to 70% confluency. Experiments were conducted to optimize and validate transfection efficiency. After serum deprivation for 24 h, HESCs were transfected with the miR mimic positive control (25 pmol/well, mirVana miRNA Mimic miR-1 Positive Control) or the miR inhibitor positive control (25 pmol/well, mirVana miRNA Inhibitor let-7c Positive Control) at three doses of Lipofectamine RNAiMAX (29 µL/mL, 60 µL/mL or 128 µL/mL) (Supplementary Fig. 1). Media were changed at 24 h and cells harvested 48 h after transfection for RNA analysis. Additional transfection experiments with miR-27b mimic and miR-27b inhibitor were performed with 29 µL/mL Lipofectamine RNAiMAX for 48 h.

### *In vitro* decidualization

HESCs were plated at 3 × 10^5^ cells/well in six-well plates in DMEM:F12 phenol red-free media and allowed to adhere to the plates. At 24 h, the media was changed to include 0.1% EtOH (vehicle) or 1 μM medroxyprogesterone acetate and 0.5 mM dibutyryl cAMP (decidualization media). The treatment media were changed every third day until cell harvest after 8 days.

### VEGF-B ELISA

HESCs were plated in six-well plates and grown to confluence in DMEM:F12 phenol red-free media with 10% charcoal-stripped serum. Thereafter, cells were rinsed and treated with vehicle (0.1% EtOH) or E2 (3.6 nM) in 1.0 mL of serum-free media in triplicate for 4 days. To ensure that E2 remained active, E2 or vehicle was added to the media every 24 h. VEGF-B protein was quantified in the media using the human VEGFB ELISA Kit (LSBIO, catalog no. LS-F5203, Seattle, WA, USA) according to the manufacturer’s instructions. Each experiment was calibrated with the standard amounts of known protein in blank media, and linear concentration-dependent curves were obtained. Each sample was measured in duplicate.

### Statistical analysis

Analyses were performed with GraphPad Prism, version 6. Each experiment was performed in triplicate on multiple cell preps as noted in figure legends. Student’s *T*-test, ANOVA with Tukey’s or Dunn’s *post hoc* analyses were used as appropriate to make comparisons among treatment groups or with controls. *P* values ≤0.05 were considered statistically significant. Statistical analysis of microarray data is described above.

## Results

### Multiple miRs are differentially regulated by hormonal treatments

To initiate discovery of potential hormonally regulated miRs in endometrial stromal cells, HESCs were treated with vehicle, 3 nM E2, 100 nM P4 or E2 (3 nM) + P4 (100 nM) for 16 h and microarray analysis was conducted. A heat map of selected differentially expressed microRNAs is shown in Supplementary Fig. 2A, and comprehensive lists of differentially expressed miRs with hormone treatment are provided in Supplementary Tables 1, 2 and 3.

### Reference microRNA validation

To confirm the microarray findings, validation of reference microRNAs was required. Microarray analysis identified miRs with stable expression in HESCs (Supplementary Fig. 2B). NormFinder (https://moma.dk/normfinder-software) indicated that the most stable miR across all treatment groups was miR-221-3p (Table 1). Four additional candidate reference microRNAs (miR-26a-5p, miR-186-5p, miR-191-5p and miR-361-5p) were selected based on stability in other reproductive tissues or per recommendations from Applied Biosystems (Shen *et al.* 2011). Expression of the miRs in HESCs was analyzed by RT-qPCR followed by NormFinder (Table 1). Hormonal treatment did not affect expression of these miRs. Because miR-26a-5p, miR-191-5p and miR-221-3p were the most highly expressed and stable in all experiments, these miRs were validated (Table 1) and used as reference miRs for TaqMan Advanced miR assays. Specifically, expression of the target miR was normalized to the mean of miR-26a-5p, miR-191-5p and miR-221-3p using the δ-δ Ct method.

**Table 1:** NormFinder stability indexes in human endometrial stromal cells.

miR	Stability value		
	Microarray primary HESCs	qPCR primary HESCs	qPCR cell line T-HESCs
26a-5p	0.224	0.002	0.005
186-5p	N.D.	0.006	N.D.
191-5p	0.160	0.002	0.007
221-3p	0.086	0.020	0.011
361-5p	N.D.	0.005	0.007

HESCs, human endometrial stromal cells; N.D., not done.

### miR-27b, miR-181b and let-7c are affected by E2 and BPA

**Figure 1:**
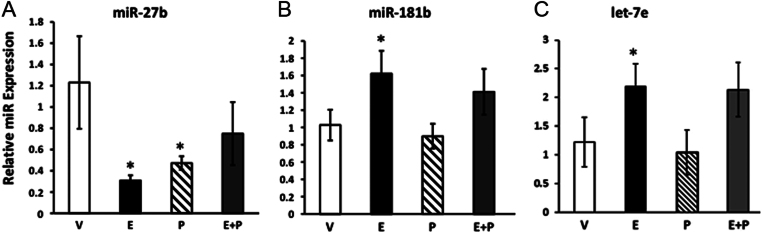
Effect of estrogen and progesterone on miR expression in HESCs. Cells were treated with vehicle (V), estradiol (E, 3 nM), progesterone (P, 100 nM) or E + P for 24 h. Data represent mean ± s.e.m. of representative experiment conducted in triplicate and replicated in an additional cell prep. *P < 0.05 compared with control, ANOVA with Dunnett’s *post hoc* analysis.

To confirm our microarray findings, we selected miRs that were highly expressed, highly conserved and significantly different from vehicle in hormone-treated cells (E2, P4 or both). These criteria, together with biological plausibility, led us to analyze miR-27b-3p, miR-181b-5p, miR-3613-3p, let-7c-5p, let-7e-5p and let-7f-5p. Because miR-3613 and let-7f were not consistently expressed in endometrial stromal cells, they were not analyzed in subsequent experiments. Whereas both E2 and P4 downregulated miR-27b, miR-181b and let-7e were upregulated by E2, but not P4 (Fig. 1). The results, therefore, were not completely congruous with microarray results in which progesterone upregulated let-7e. Thus, we focused the investigation on estrogen-responsive miRNAs and conducted pathway analysis for genes regulated by E2-responsive miRNAs. The top nine biological processes are listed in Table 2. Interestingly, the predominant biological process predicted to be greatly enriched by estrogen-regulated miRNAs was regulation of endothelial cell migration (18–21 fold, Table 2).

**Table 2:** Enrichment analysis of predicted biological processes affected by estrogen-regulated microRNAs.

Biological process term	%	*P* value	Fold enrichment
GO:0045449~regulation of transcription	25.17	6.40E-07	1.75
GO:0006350~transcription	19.58	6.50E-05	1.68
GO:0051252~regulation of RNA metabolic process	16.43	6.14E-04	1.64
GO:0010596~regulation of endothelial cell migration	1.40	1.23E-03	18.06
GO:0006355~regulation of transcription, DNA-dependent	15.38	2.34E-03	1.57
GO:0006357~regulation of transcription from RNA polymerase II promoter	7.69	5.34E-03	1.91
GO:0007242~intracellular signaling cascade	11.19	7.89E-03	1.61
GO:0043537~regulation of blood vessel endothelial cell migration	1.05	8.26E-03	21.07
GO:0008219~cell death	7.34	9.67E-03	1.85

% represents involved genes/total genes. *P* value represents EASE Score, a modified Fisher Exact *P* value to measure the probability of Count/List total) is more than random compared with background list.

**Figure 2:**
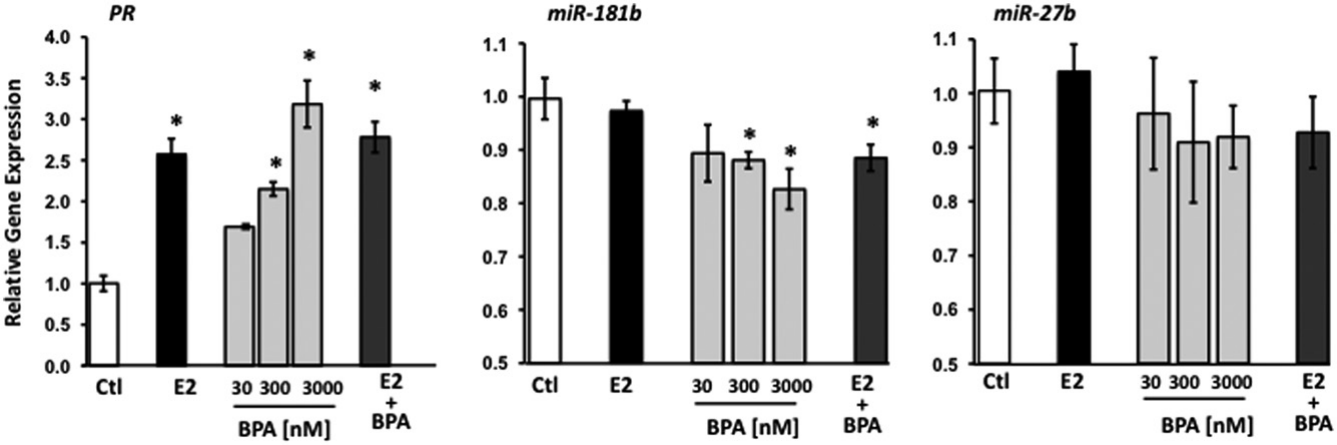
BPA dose-dependently regulates PR and miR-181b gene expression. HESCs were treated with vehicle (Ctl), E2 (3 nM), BPA (30–3000 nM) or E2 3 nM and BPA 3000 nM for 24 h. Data represent mean ± s.e.m. of representative experiment conducted in triplicate and replicated in an additional cell prep. *P < 0.05 compared with control, ANOVA with Dunnett’s *post hoc* analysis.

**Figure 3:**
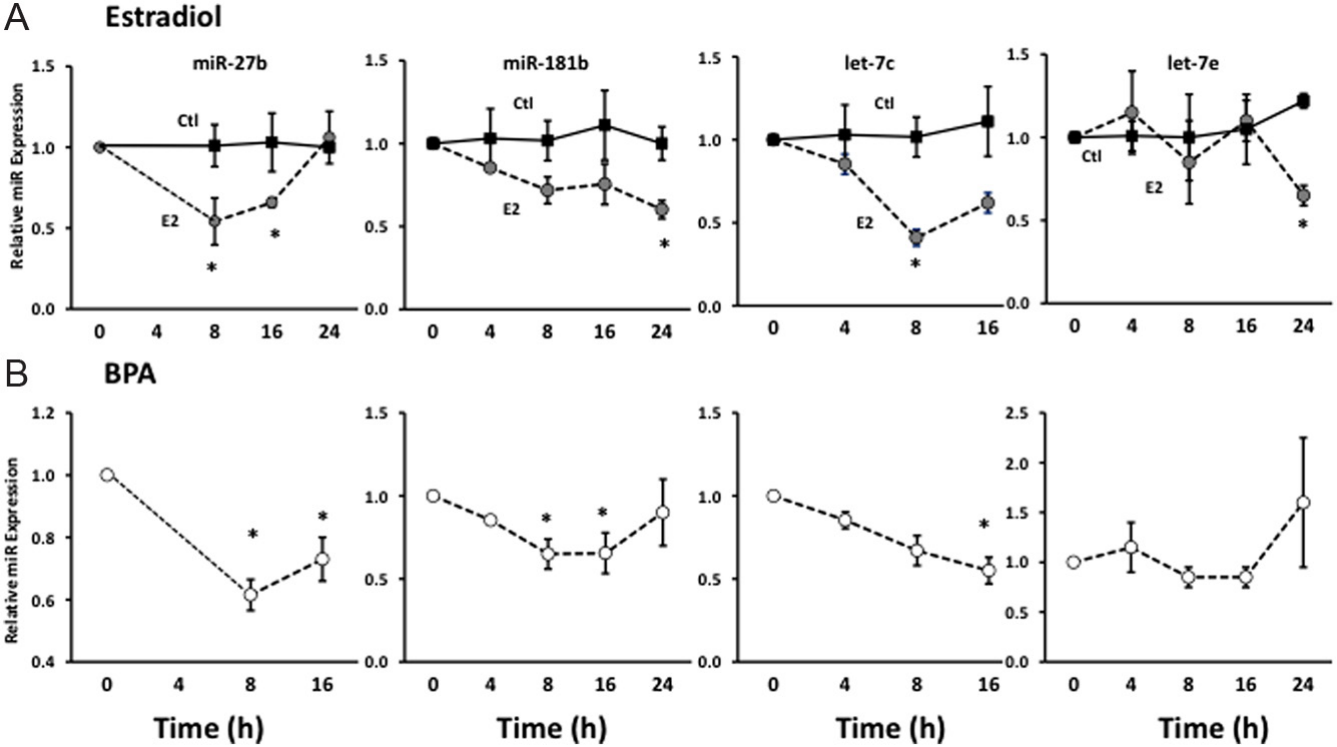
E2- and BPA-induced regulation of miR-27b, -181b, let-7c and let-7e. HESCs were treated with E2 (3 nM) (A) or BPA (B) as a function of time. RNA was isolated at baseline or various times after treatment (4–24 h). Panel A depicts treatment with vehicle (Ctl, black squares) or E2 (circles). Panel B represents data after treatment with BPA (3 µM). Data represent mean ± s.e.m. of representative experiments conducted in triplicate and repeated in six cell preps except let-7c, which was conducted in one cell prep. *P < 0.05 compared with baseline, ANOVA with Dunnett’s *post hoc* testing using time 0 as the control.

Serum concentrations of BPA in human subjects range from 1 to 10 ng/mL although higher concentrations may be found in other biological fluids such as amniotic fluid. Hence, our initial dose response with BPA was conducted from 30 nM (6 ng/mL) to 3 µM for 24 h. Progesterone receptor (PR) gene expression was used as a classic estrogen-responsive gene in HESCs. Thus, cells were treated with E2 (3 nM), BPA (30–3000 nM) or E2 (3 nM) + BPA (3 µM) for 24 h. Thereafter, RT-qPCR was performed to validate selected miRs and PR as an index of estrogen responsiveness (Fig. 2). BPA, at concentrations of 300 nM, increased PR gene expression and downregulated miR-181b. miR-27b was not regulated by E2 or BPA at 24 h. These experiments indicate that the cells are responsive to BPA and BPA does not appear to have additive effects with E2. Similar to other investigations in cell culture systems (Dairkee *et al.* 2013, Hwang *et al.* 2013, Dong *et al.* 2014, Chen *et al.* 2015, Nakano *et al.* 2016), further experiments were conducted with 3 µM BPA. The lack of BPA-induced regulation of miR-27b at 24 h, although consistent with microarray data, led us to conduct temporal relationships between E2- and BPA-induced regulation of miR-27b, miR-181b, let-7c and let-7e (Fig. 3).

All experiments showed no change in any miR with vehicle alone as a function of time in culture (Fig. 3A, Ctl). E2, however, resulted in time-dependent regulation of four miRs, but the time course varied thereby explaining the lack of response of some miRs at 24 h (Fig. 3A). The time course of BPA-induced regulation of miR-27b, -181b, let-7c and let-7e was compared with that of E2 (Fig. 3A and B). Both E2 and BPA downregulated miR-27b expression in the 8–16 h time frame. BPA also resulted in significant downregulation of miR-181b from 8 to 16 h with return to normal levels by 24 h. Let-7c was downregulated by E2 and BPA, but the time course differed between E2 and BPA (Fig. 3). Let-7e was most significantly affected in the E2 treatment group at the 24-h time point. Taken together, these data indicate that E2 (in physiological concentrations) and BPA time dependently regulate expression of miR-27b, miR-181b and let-7c. In addition, let-7e is affected by E2, but not BPA during this treatment timeframe.

### Potential targets of miR-27b: VEGFB and VEGFC

**Figure 4:**
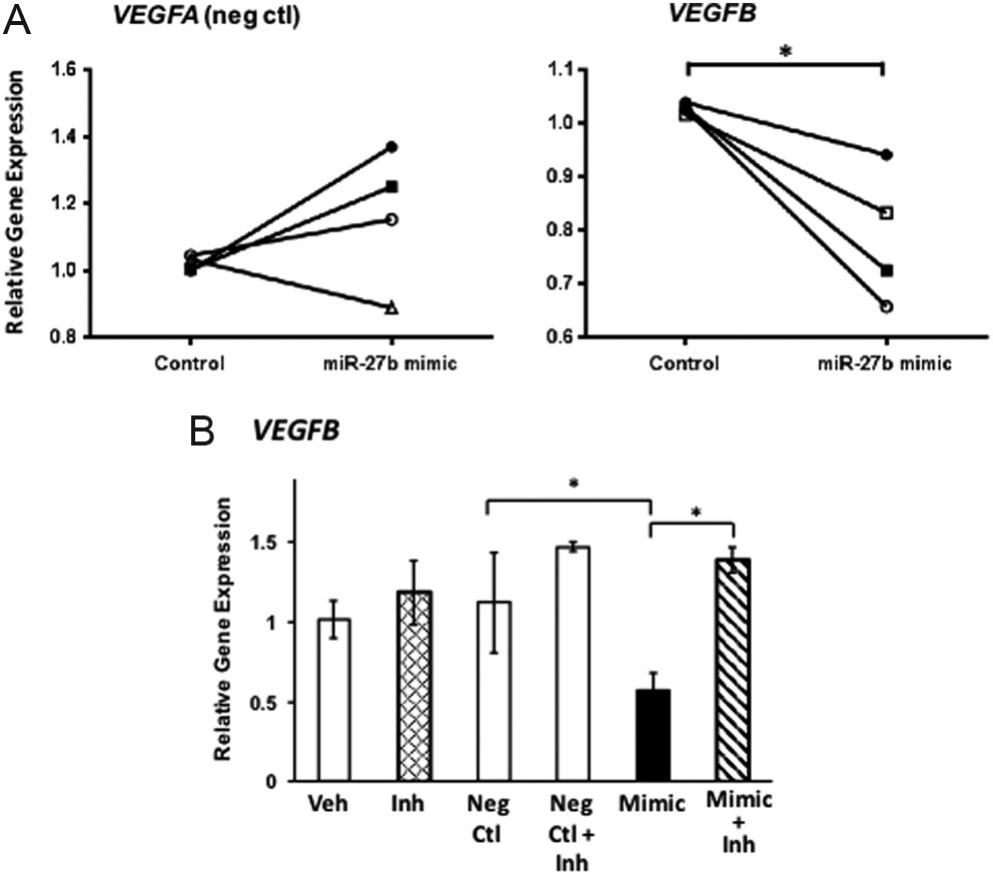
*VEGFB* is a target of miR-27b in human endometrial stromal cells. (A) HESCs were transfected with negative control or miR-27b mimic for 48 h in cell preps from four different patients. Gene expression of *VEGFA* (negative control), and *VEGFB* were quantified by qPCR. Each symbol represents mean values of triplicates from each cell prep. *P < 0.05, paired *t*-test. (B) Cells were treated with vehicle, miR-27b inhibitor, negative control, negative control + inhibitor, miR-27b mimic, or miR-27b mimic + inhibitor for 48 h. Thereafter, relative mRNA levels of *VEGFB* were determined. *P < 0.05, ANOVA.

**Figure 5:**
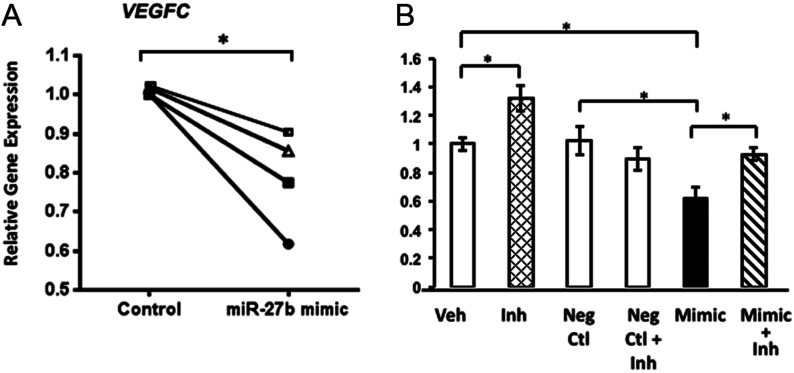
*VEGFC* is a target of miR-27b in human endometrial stromal cells. (A) HESCs were transfected with negative control or miR-27b mimic for 48 h in cell preps from four different patients. Gene expression of *VEGFC* was quantified by qPCR. Each symbol represents mean values of triplicates from each cell prep. *P < 0.05, paired *t*-test. (B) Cells were treated with vehicle, miR-27b inhibitor, negative control, negative control + inhibitor, miR-27b mimic, or miR-27b mimic + inhibitor for 48 h. Thereafter, relative mRNA levels of *VEGFC* were determined. *P < 0.05, ANOVA.

Next, we sought to identify miR-27b targets and the potential physiological relevance of E2- and BPA-induced regulation of miR-27b. This particular miR was chosen because (i) the magnitude of suppression and time course of suppression at 8 and 16 h by E2 and BPA were similar and (ii) miR-27b has been shown to be important for other tissues that undergo significant remodeling and vascularization (Kang *et al.* 2013, Chen *et al.* 2017*b*). TargetScan (www.targetscan.org) was used to screen potential miR-27b targets. Several mRNA targets (estrogen receptor α, *ERα*; estrogen receptor β, *ERβ*; progesterone receptor, *PGR*; Forkhead box O3, *FOXO3*; Forkhead box O1, *FOXO1*; insulin-like growth factor-binding protein 1, *IGFBP1*; vascular endothelial growth factors, *VEGFA*, *VEGFB*, *VEGFC*, *VEGFD*, and Homeobox A10, *HOXA10A*) were analyzed in HESCs treated with or without BPA. Of these, most were not consistent with regulation by miR-27b at the transcriptional level (not shown), or, in the case of* VEGFD*, were poorly expressed. *VEGFB* and *VEGFC*, but not *VEGFA*, demonstrated patterns compatible with potential miR-27b targets. To test if *VEGFB* was regulated by miR-27b in HESCs, cells were transfected with non-targeting, miR-27b mimic or miR-27b inhibitor oligomers. Consistent with our experiments demonstrating no effect of BPA, *VEGFA* was not regulated by miR-27b (Fig. 4A). *VEGFB* gene expression, however, was decreased significantly by miR-27b mimic in four cell preps from different patients (Fig. 4A). Although gene expression was not increased by inhibitor alone, the inhibitor reversed miR-27b mimic-induced downregulation of *VEGFB* (Fig. 4B). Likewise, miR-27b mimic downregulated *VEGFC* significantly (Fig. 5A). Whereas miR-27b inhibitor upregulated, miR-27b mimic downregulated VEGFC 50%, and the inhibitor reversed its downregulation (Fig. 5B).

**Figure 6:**
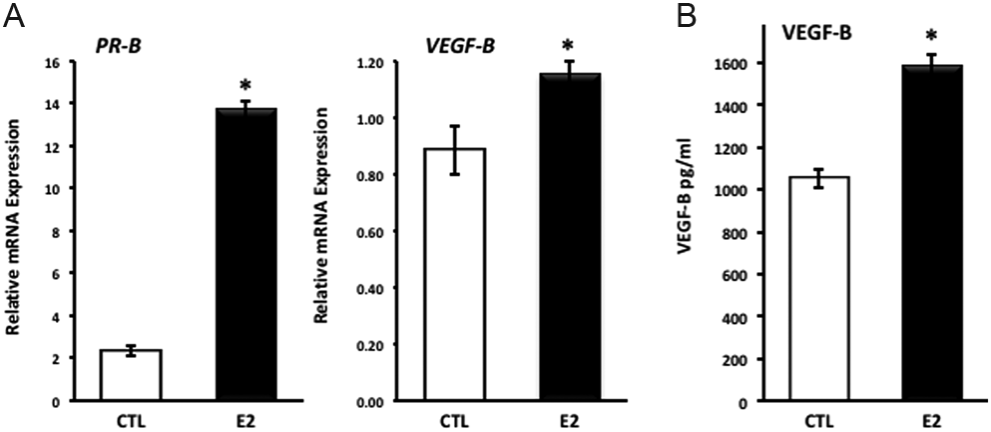
E2 regulates VEGF-B mRNA and protein. HESCs were treated with vehicle (CTL) or E2 (3.6 nM) for 4 days. (A) To confirm estrogen responsiveness, *PR-B* and *VEGF-B* mRNA was quantified. VEGF-B protein was quantified in media. *P < 0.05, Students *t*-test.

To confirm that these targets were upregulated at the protein level, we analyzed VEGF-C and VEGF-B protein in control and E2-treated cells. The amount of VEGF-C protein was too low to detect reliably in stromal cell conditioned media despite concentrating the media five-fold. On the other hand, VEGF-B was readily detectable. The experiment was conducted after 4-day treatment to optimize the accumulation of the protein. Treatment of HESCs with E2 (3.6 nM) for 4 days resulted in six-fold increases in the estrogen-responsive gene, PR-B (Fig. 6A). E2 treatment resulted in modest, but significant, increases in VEGF-B gene expression (Fig. 6A) and proportionate increases in VEGF-B accumulation in the media (Fig. 6B). Taken together, *VEGFB* is a target of miR-27b in endometrial stromal cells. These findings are compatible with our pathway analysis of microarray data showing estrogen-induced enrichment of endothelial cell migration (Table 2).

### miR-27b expression is downregulated in decidualized cells

**Figure 7:**
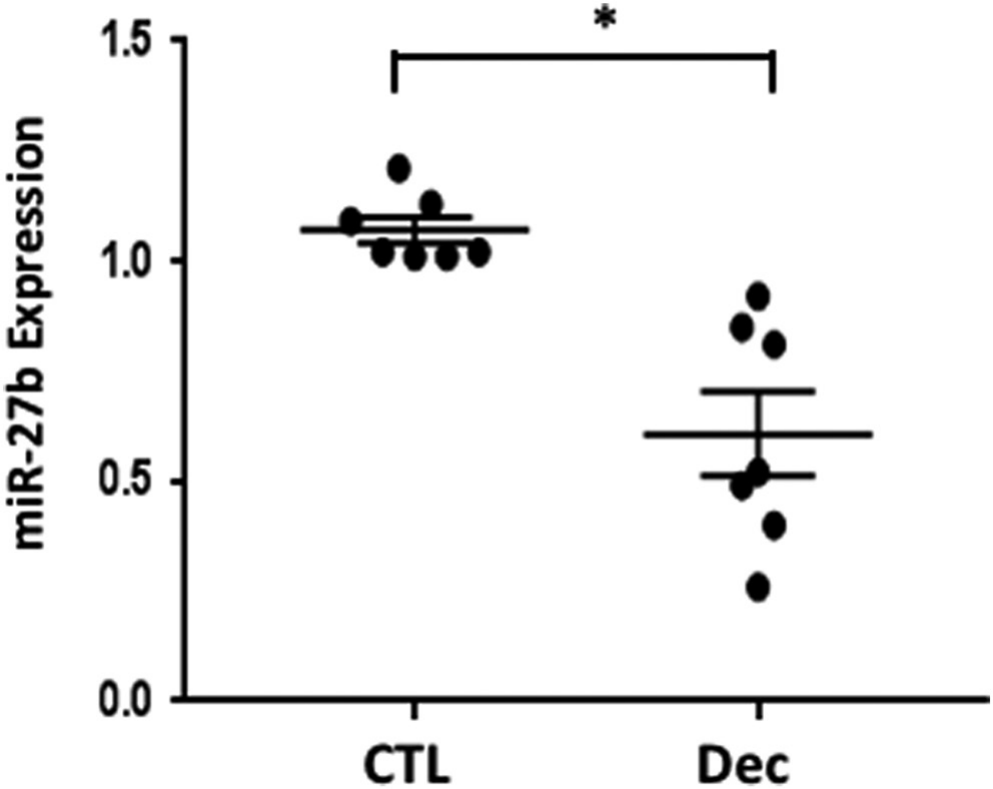
*In vitro* decidualization downregulates miR-27b. HESCs were decidualized *in vitro* for 8 days. miR-27b expression was quantified by qPCR. Data represent mean values of seven cell preps conducted in triplicate. *P < 0.05, paired *t*-test.

To determine if miR-27b expression is altered during decidualization, HESCs were treated with dibutyryl cAMP and medroxyprogesterone acetate for 8 days. Decidualization was assessed by increased expression of *PRL* and *IGFBP1* mRNA and morphological change in the cells (not shown). Interestingly, miR-27b was downregulated with decidualization (Fig. 7) in seven different cell preps.

## Discussion

Several investigations have profiled miRNA expression in secretory and proliferative endometrium (Kuokkanen *et al.* 2010, Wessels *et al.* 2013, Dior *et al.* 2014) and differences in miRNA expression in spontaneous decidualized endometrium and early pregnancy decidua (Wang *et al.* 2016). In some studies, the endometrium was considered as a whole tissue (Ohlsson Teague *et al.* 2009), whereas in others, endometrial epithelial cells were targeted. It is thereby difficult to ascertain the cell type of miR expression and bona fide targets within the cell type of origin or adjacent cells. Here, we hypothesized that various miRNAs may play a role in estrogen- and progesterone-induced development of the endometrial stroma. Further, we speculated that the hormone disruptor BPA may alter endometrial miR gene expression.

Microarray analysis of HESCs treated with E2 and/or P4 supports the differential expression of many miRs in human endometrial stromal cells with exposure to E2. Because the microarray only examined expression of miRs at a single point in time and in a single patient, it served as a springboard to investigate temporal relationships between estradiol, miR expression and regulation in primary cell cultures. We show for the first time that miR-27b is downregulated by E2 and BPA in endometrial stromal cells, and we identified miR-27b targets in these cells. The results are in agreement with Wang *et al.* in which E2 downregulated miR-27b in a leukemia cell line (Wang *et al.* 2014) and with Ye *et al.* in which *VEGFC* was shown to be a validated target of miR-27b in 293T and colorectal cancer cells (Ye *et al.* 2013).

### Physiological relevance of miR-27b

miR-27b is often considered a tumor suppressor. It has been shown to be downregulated in several cancers, including colorectal cancer and neuroblastoma (Lee *et al.* 2012, Ye *et al.* 2013). It has been suggested that miR-27b may inhibit tumor cell proliferation, migration and invasion. Our results indicate that one potential mechanism by which miR-27b may regulate these processes is through regulation of VEGFC (Ye *et al.* 2013). As miR-27b is lost in cancer cells, VEGFC expression increases, resulting in increased metastatic potential. VEGFC induces new blood vessel formation and endothelial cell proliferation and migration (Van Trappen & Pepper 2005). Further, our results support a role for miR-27b in regulation of VEGF-B.

Invasion of the blastocyst into the endometrium during pregnancy implantation has many similarities with invasion of cancer cells. We show that miR-27b is decreased during decidualization. It is known that VEGF production increases during decidualization and embryo implantation. Our results are compatible, therefore, with the possible involvement of miR-27b in regulating VEGFB and C and thereby new blood vessel formation and endothelial cell proliferation and migration during decidualization. Furthermore, VEGFs are E2 responsive and E2 is required for the initial increase of VEGFs that occur in the mid- to late-proliferative phase of the menstrual cycle (Nayak & Brenner 2002).

### BPA regulates miR-27b

BPA exposure is associated with increased vascular tube formation and branching points, as well as increased *VEGFD* mRNA and protein in human endometrial endothelial cells that also express ERβ (Helmestam *et al.* 2014). These findings suggest that BPA may alter normal vasculogenesis in the endometrium and impact its development and possibly embryo implantation. BPA also impairs placentation (Lan *et al.* 2017). Recently, BPA has been shown to alter endometrial stromal cell decidualization *in vitro*. Olson *et al.* used physiologic and supraphysiologic concentrations of BPA during *in vitro* decidualization for 8 days (Olson *et al.* 2017). They found that 10 µg/mL and 20 µg/mL were required to prevent decidualization, proliferation and alter *ERα*, *PGR* and cell cycle gene expression. The authors note that although these concentrations are greater than typical human exposure, the data support the impact of BPA on reproductive tissues.

Our data further support that BPA has a negative impact on the endometrium. Our initial experiments do not suggest an additive effect of BPA and E2; however, the impact *in vivo* is unclear. Nonetheless, exposure to BPA during the early proliferative phase when E2 levels are low may alter the timing of downregulation of miR-27b, and therefore, increase expression of its targets, *VEGFB* and *VEGFC*, prematurely leading to dysregulation of angiogenesis. The progressive and orderly development of the endometrium is well established and disruption in the process by BPA may contribute to the development of gynecologic disorders involving increased angiogenesis and VEGF gene expression in conditions such as endometriosis (Oosterlynck *et al.* 1993, McLaren *et al.* 1996), abnormal decidualization or implantation failure.

### Summary

The strengths of this study include the use of multiple cell preps of endometrial stromal cells in primary culture to ensure as much conformity as possible, and time course investigations demonstrating that single time point microarrays may be misleading. Further, the results were validated using stably expressed microRNAs as reference genes. To narrow the scope of the investigation, we focused on E2- and BPA-induced regulation of miRs. One caveat is that miRs have the ability to affect targets at the mRNA or the protein level. Here, we show that E2 induced downregulation of miR-27b and also led to increased secretion of the miR-27b target, VEGF-B. Other translational targets were not investigated. Further, we did not conduct a comprehensive evaluation of these miRs during combination treatment (BPA+E2) or endometrial decidualization, studies which are now ongoing. Nevertheless, our data indicate that BPA and E2 affect miR expression in endometrial stromal cells and may impact targets important for endometrial development. Identification of *VEGFB* and *VEGFC* as miR-27b target genes suggests that BPA and E2 alter genes important for vascularization and angiogenesis of the endometrium during the menstrual cycle, decidualization or ectopic implantation.

## Supplementary Material

Supporting Table 1

Supporting Table 2

Supporting Table 3

## Declaration of interest

The authors declare that there is no conflict of interest that could be perceived as prejudicing the impartiality of the research reported.

## Funding

This work was supported by the Human Biological Fluids Tissue Acquisition Laboratory (NIH P01HD087150) for endometrial specimens. The research was supported by grant K12 HD000849 (P T J), awarded to the Reproductive Scientist Development Program by the Eunice Kennedy Shriver National Institute of Child Health and Human Development and by the Burroughs Wellcome Fund, as part of the Reproductive Scientist Development Program.
