# Supplementary material for: Estrogen-regulated miRNA-27b is altered by bisphenol A in human endometrial stromal cells
Source: Reproduction. 2018 Sep 28;156(6):559–67. doi: 10.1530/REP-18-0041 (PMC6215928; doi:10.1530/REP-18-0041)
Supplement: Supporting Table 1 [file rep-156-559-t001.pdf]

sTable 1. Differential expression of microRNAs between vehicle and estradiol-treated (E2) cells at 16 h. Data represent mean signal intensities of vehicle- or estradiol-treated cells. Transcripts statistically significant but of low signal intensity (<500 by microarray) are not shown.

MicroRNA	p-value	Vehicle Mean	Estradiol Mean	Log2 (E2/Veh)
hsa-miR-1260b	1.81E-03	5,325	4,401	-0.27
hsa-miR-4280	2.88E-03	24	669	4.81
hsa-miR-6865-5p	3.21E-03	101	1,187	3.56
hsa-miR-6820-5p	3.71E-03	85	1,613	4.25
hsa-miR-7843-5p	4.03E-03	8	756	6.51
hsa-miR-6085	6.40E-03	743	1,042	0.49
hsa-miR-4497	1.51E-02	4,633	6,024	0.38
hsa-miR-6779-5p	1.62E-02	363	614	0.76
hsa-miR-4530	1.74E-02	1,706	2,308	0.44
hsa-miR-3620-5p	1.85E-02	378	585	0.63
hsa-miR-6753-5p	2.09E-02	122	716	2.55
hsa-miR-4505	2.66E-02	665	1,139	0.78
hsa-miR-20b-5p	2.89E-02	1,153	906	-0.35
hsa-miR-1910-3p	3.19E-02	32	596	4.23
hsa-miR-197-3p	4.42E-02	606	383	-0.66
hsa-miR-320d	5.54E-02	1,211	1,462	0.27
hsa-miR-548m	5.66E-02	40	616	3.95
hsa-miR-498	5.88E-02	62	514	3.06
hsa-miR-4701-5p	6.24E-02	626	259	-1.27
hsa-miR-149-3p	6.84E-02	533	663	0.32
hsa-miR-6087	7.21E-02	7,268	8,338	0.20
hsa-miR-423-5p	8.22E-02	490	1,086	1.15
hsa-miR-1273g-3p	8.72E-02	6,180	7,387	0.26
