# Supplementary material for: Estrogen-regulated miRNA-27b is altered by bisphenol A in human endometrial stromal cells
Source: Reproduction. 2018 Sep 28;156(6):559–67. doi: 10.1530/REP-18-0041 (PMC6215928; doi:10.1530/REP-18-0041)
Supplement: Supporting Table 2 [file rep-156-559-t002.pdf]

sTable 2. Differential expression of microRNAs between vehicle and progesterone-treated (P4) cells at 16 h. Data represent mean signal intensities of vehicle- or progesterone-treated cells. Transcripts statistically significant but of low signal intensity (<500 by microarray) are not shown.

MicroRNA	p-value	Vehicle Mean	Progesterone Mean	Log2 (P4/Veh)
hsa-miR-1273g-3p	3.91E-03	6,180	7,899	0.35
hsa-miR-361-5p	1.03E-02	1,138	937	-0.28
hsa-miR-23c	1.24E-02	6,390	7,390	0.21
hsa-let-7e-5p	1.27E-02	7,542	13,322	0.82
hsa-let-7c-5p	1.32E-02	9,913	15,986	0.69
hsa-let-7d-5p	1.40E-02	9,915	16,105	0.70
hsa-let-7f-5p	1.51E-02	9,566	16,207	0.76
hsa-miR-320d	1.63E-02	1,211	1,734	0.52
hsa-miR-7108-5p	1.75E-02	1,136	856	-0.41
hsa-miR-4301	2.11E-02	3,710	5,275	0.51
hsa-miR-148a-3p	2.23E-02	629	463	-0.44
hsa-let-7a-5p	2.36E-02	11,213	18,314	0.71
hsa-miR-4455	3.38E-02	650	1,004	0.63
hsa-miR-498	3.44E-02	62	616	3.32
hsa-miR-548m	3.52E-02	40	781	4.29
hsa-miR-4668-5p	3.59E-02	2,917	9,765	1.74
hsa-miR-27b-3p	3.66E-02	11,314	8,682	-0.38
hsa-miR-15b-5p	3.86E-02	1,890	3,080	0.70
hsa-miR-424-5p	4.52E-02	17,664	11,871	-0.57
hsa-miR-8069	4.57E-02	3,431	2,851	-0.27
hsa-miR-151a-5p	4.61E-02	2,900	2,390	-0.28
hsa-miR-1915-3p	4.86E-02	2,231	1,569	-0.51
hsa-miR-320e	4.99E-02	1,266	1,585	0.32
hsa-miR-6727-5p	5.20E-02	6,745	5,899	-0.19
hsa-miR-99b-5p	5.26E-02	3,254	2,650	-0.30
hsa-miR-186-5p	5.69E-02	906	611	-0.57
hsa-miR-148b-3p	5.89E-02	557	464	-0.26
hsa-let-7i-5p	5.97E-02	4,701	5,424	0.21
hsa-miR-335-5p	6.09E-02	5,122	2,897	-0.82
hsa-miR-6803-5p	6.15E-02	2,152	1,581	-0.45
hsa-miR-99a-5p	6.29E-02	5,840	4,031	-0.53
hsa-miR-8485	6.47E-02	3,353	5,784	0.79
hsa-miR-27a-3p	6.74E-02	9,066	6,964	-0.38
hsa-let-7b-5p	6.86E-02	7,996	11,444	0.52
hsa-miR-23b-3p	7.47E-02	13,296	16,067	0.27
hsa-miR-6743-5p	7.61E-02	2,039	1,492	-0.45
hsa-miR-23a-3p	7.90E-02	12,674	15,207	0.26
hsa-miR-320a	8.09E-02	1,585	2,274	0.52
hsa-miR-365a-3p	8.19E-02	974	1,382	0.50
hsa-miR-21-5p	8.24E-02	20,179	34,109	0.76
hsa-miR-425-5p	8.29E-02	593	382	-0.63
hsa-miR-22-5p	8.32E-02	911	603	-0.59
hsa-miR-1469	8.69E-02	604	372	-0.70
hsa-miR-3613-3p	8.75E-02	1,737	6,361	1.87
hsa-miR-423-5p	9.38E-02	490	881	0.85
hsa-miR-151b	9.61E-02	2,675	2,122	-0.33

hsa-miR-29a-3p	9.72E-02	17,554	14,814	-0.24
hsa-miR-5001-5p	9.80E-02	1,719	1,217	-0.50
hsa-miR-20b-5p	9.88E-02	1,153	698	-0.72
hsa-miR-7704	9.96E-02	6,138	5,099	-0.27
