# Supplementary material for: Estrogen-regulated miRNA-27b is altered by bisphenol A in human endometrial stromal cells
Source: Reproduction. 2018 Sep 28;156(6):559–67. doi: 10.1530/REP-18-0041 (PMC6215928; doi:10.1530/REP-18-0041)
Supplement: Supporting Table 3 [file rep-156-559-t003.pdf]

sTable 3. Differential expression of microRNAs between vehicle and Estradiol+progesterone-treated (EP) cells at 16 h. Data represent mean signal intensities of vehicle- or EP-treated cells. Transcripts statistically significant but of low signal intensity (<500 by microarray) are not shown.

MicroRNA	p-value	Vehicle Mean	EP Mean	Log2 (EP/Veh)
hsa-miR-335-5p	8.66E-03	5,122	1,601	-1.68
hsa-miR-335-3p	1.00E-02	811	277	-1.55
hsa-miR-424-5p	1.57E-02	17,664	8,745	-1.01
hsa-miR-99a-5p	1.63E-02	5,840	3,581	-0.71
hsa-miR-4508	2.01E-02	1,540	2,917	0.92
hsa-miR-4497	2.19E-02	4,633	6,952	0.59
hsa-miR-4787-5p	2.54E-02	4,551	7,275	0.68
hsa-miR-181b-5p	2.54E-02	2,586	1,641	-0.66
hsa-miR-145-3p	3.49E-02	654	395	-0.73
hsa-miR-4488	3.80E-02	4,931	7,119	0.53
hsa-miR-1260b	4.70E-02	5,325	3,284	-0.70
hsa-miR-181d-5p	4.76E-02	1,892	613	-1.63
hsa-miR-10a-5p	5.07E-02	569	249	-1.19
hsa-miR-4324	5.14E-02	17,401	10,199	-0.77
hsa-miR-4532	5.27E-02	417	571	0.45
hsa-miR-100-5p	5.41E-02	7,446	4,722	-0.66
hsa-miR-765	5.43E-02	760	468	-0.70
hsa-miR-320d	5.49E-02	1,211	2,060	0.77
hsa-miR-423-5p	6.89E-02	490	959	0.97
hsa-miR-27a-3p	6.90E-02	9,066	5,897	-0.62
hsa-miR-365a-3p	7.42E-02	974	554	-0.81
hsa-miR-3178	7.80E-02	3,146	4,835	0.62
hsa-miR-450a-5p	8.11E-02	852	408	-1.06
hsa-miR-29c-3p	8.59E-02	6,975	2,211	-1.66
hsa-miR-6769a-5p	8.96E-02	515	343	-0.59
hsa-miR-148a-3p	9.58E-02	629	414	-0.60
